# Supplementary material for: Correlation between social proximity and mobility similarity
Source: Sci Rep. 2017 Sep 20;7:11975. doi: 10.1038/s41598-017-12274-x (PMC5607281; doi:10.1038/s41598-017-12274-x)
Supplement: Supplementary file 1 — Supporting Information [file 41598_2017_12274_MOESM1_ESM.pdf]

Supporting Information

for "Correlation between social proximity and mobility similarity"
by Chao Fan, Yiding Liu, Junming Huang, Zhihai Rong and Tao Zhou

1 Probability distributions

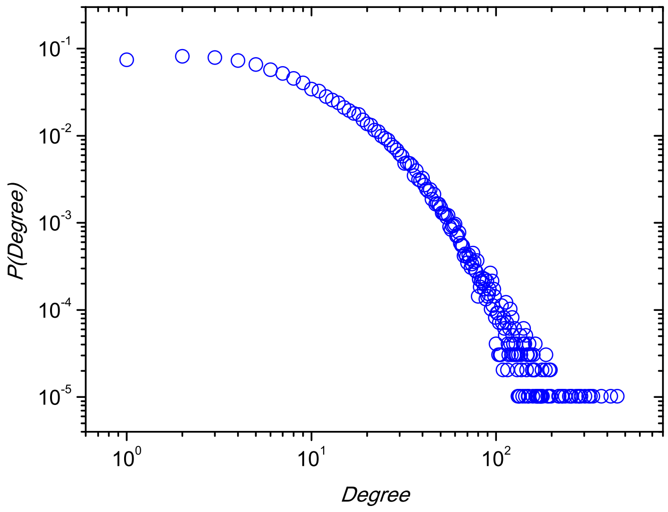

Figure S1: **The degree distribution of online social network.** It shows a heavy-tail shape which can be fitted by the function of power-law with exponential cutoff as: $f(x) \propto x^a * e^{-bx}$, where $a = 0.157 \pm 0.027, b = 0.121 \pm 0.005$..

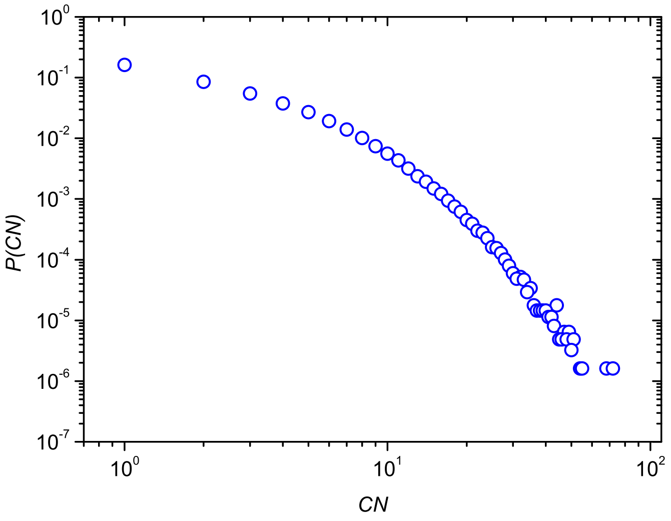

Figure S2: **The distributions of *CN*.** *CN* follows the distribution of power-law with exponential cutoff as $f(x) \propto x^a * e^{-bx}$, where $a = -0.595 \pm 0.012, b = 0.214 \pm 0.005$.

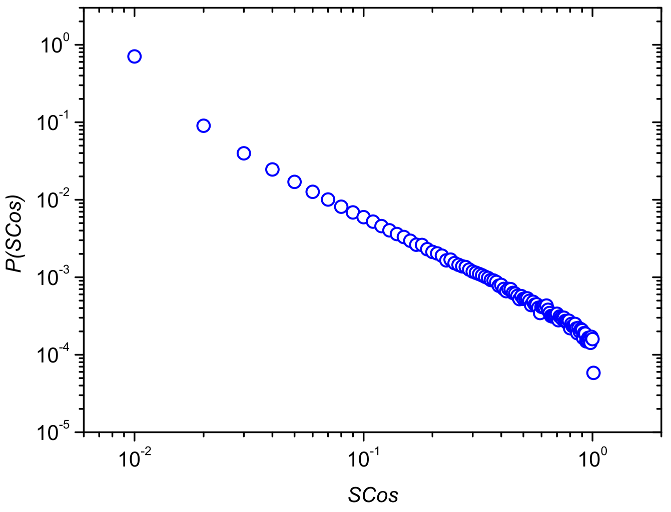

Figure S3: **The distributions of *SCos*.** The distribution of *SCos* behaves power-law shape as $f(x) \propto x^a$, where $a = -1.041 \pm 0.003$. We add 0.01 for each data point of *SCos* to better illustrate the zero values.

2 Null model

A maximal random network of randomly shuffled edges provides a baseline to compare the correlation between friends and strangers, further highlight the significance of social relations. Accordingly, we take the following steps to do the experiments [37-40]: (1) switch two randomly selected real edges, i.e., unlink (A,B) and (C,D) and then link (A,C) and (B,D), on the premise that (A,C) and (B,D) are not actually connected; (2) repeat the step $10 * N$ times, where N is the number of edges in the network; (3) calculate the $SCos$ and CN of each new pair of friends; (4) calculate the mean $SCos$ with and without common friends; (5) take out 50 parallel experiments and report the mean value and standard deviation of the average $SCos$.

Through this method we obtain a null model without changing the degree sequence of the original network and give a benchmark to compare the significant findings of our manuscript. The results show that the average $SCos$ of pairs in the null model (0.00078 ± 1.5e-05) is significantly lower than that between real friends (0.03084), approaching that of non-friends (0.00049). Therefore we claim that shuffling social relations significantly decreases the similarity between individuals. Besides, the average $SCos$ of group $CN > 0$ and $CN = 0$ are 0.00238 ± 3.1e-04 and 0.00074 ± 1.5e-05 respectively, still higher than that of non-friends.

Table S1 give a comparison of the average $SCos$ of real data and null model. It can be observed that the average $SCos$ decreases by two orders of magnitude in null model compared with real data, indicating that the real social relationship could promote the mobility similarity between two individuals. Besides, the $SCos$ distributions of the two groups with $CN > 0$ and $CN = 0$ are shown in Fig. S4. In a word, the mobility similarity among shuffled friends is lower than real friends and higher than non-friends. The impacts of being friends and having common friends which we observed in real data are significant.

Table S1: **Comparison of average $SCos$ between real data and null model**

Average $SCos$	All pairs of friends	Groups of $CN > 0$	Groups of $CN > 0$	Non-friends
Real data	0.03084	0.04149	0.00810	0.00049
Null model	0.00078±1.5e-05	0.00238±3.1e-04	0.00074±1.5e-05	—

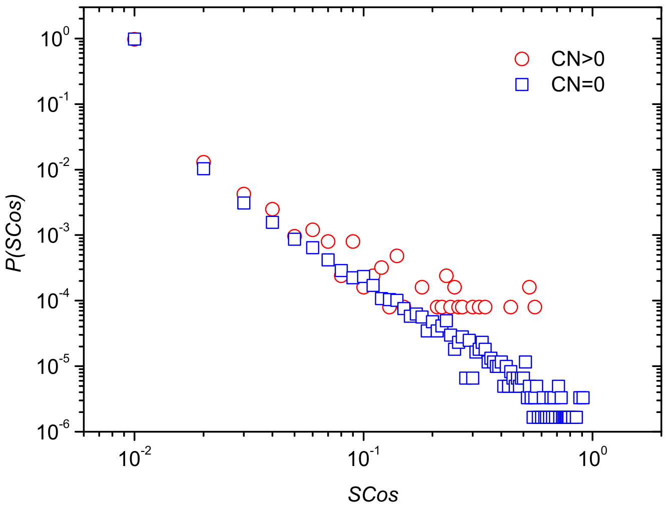

Figure S4: **The *SCos* distributions of pairs with and without common friends in null model by rewiring edges.** The red circles and blue squares represent the groups with $CN > 0$ and $CN = 0$ respectively. We add 0.01 to each data point to better illustrate zero in a log-log plot.

3 KS test

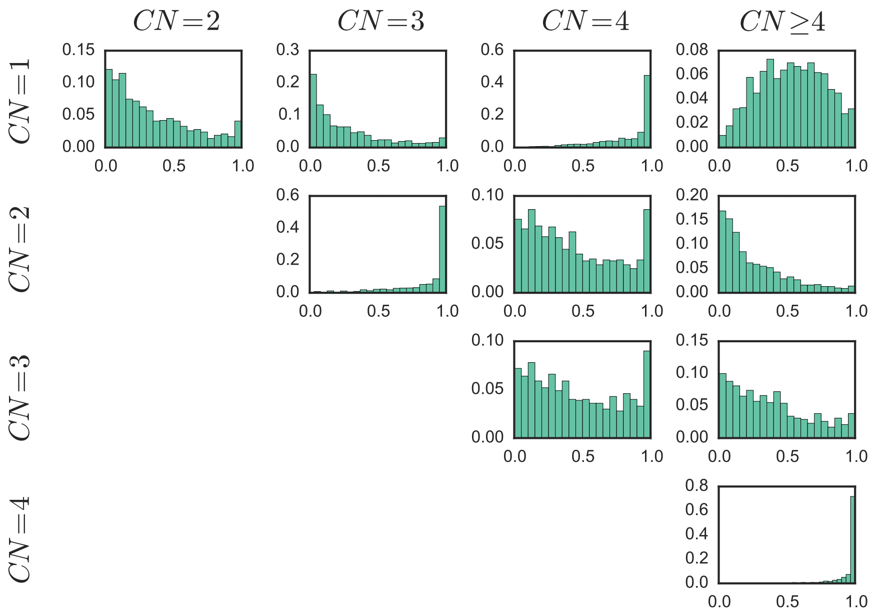

Figure S5: **The distributions of *p-value* of each KS test between groups with different *CN*.** It's clear that all the distributions are not normally distributed, indicating that all pair of two distributions with different CN are drawn from the same population.

4 Co-location rate

Taking temporal factor into consideration, we choose *Co-location rate (CoL)* as an alternative similarity measure of *SCos*. The index *CoL* measures the probability of two individuals going to the same destination at the same time, which is defined as [28]:

$$CoL(x,y) = \frac{\sum_{i=1}^{n(x)} \sum_{j=1}^{n(y)} \Theta(\Delta T - |T_i(x) - T_j(y)|)\delta(L_i(x), L_j(y))}{\sum_{i=1}^{n(x)} \sum_{j=1}^{n(y)} \Theta(\Delta T - |T_i(x) - T_j(y)|)},$$

where $\Theta(x)$ is the Heaviside step function, ΔT is the inter-event time between two check-ins of different individuals, $\delta(a,b) = 1(a = b)$ or $0(a \neq b)$, $L_i(x)$ is the i-th location individual x has visited. We set 1 km and 0.5h as threshold to determine whether the co-occurrence is at the same location and within a specific time window. Like $SCos$, CoL also captures how similar two individuals' visiting patterns are.

Based on this definition, we calculate the CoL of every pair of individuals and take out the same experiments as that in main text. The semilog coordinates plot in Fig. S6(c) shows that the CoL follows exponential distribution. Figure S6(a) and (b) indicate that friends (or friends with common neighbors) behave more similarly than non-friends (or friends without common neighbors). Befriending and having common friends are mutually independent factors (Fig. S6(d)). When CN is controlled, mobility similarity increases with CC, while CN has no positive impact on similarity when CC is controlled (Fig. S6(e) and (f)).

5 Connectivity

The *connectivity* of a connected graph G is defined as the minimum number of nodes or edges whose removal would disconnect G [45,46]. For specific nodes s and t, the *node- (or edge-) connectivity* for two distinct nodes s and t denote the minimum number of vertices (or links) which must be removed to destroy all paths from s to t in G. So *connectivit* could not only measure the reachability between tow nodes but also count the number of independent pathways between them. If there are many independent pathways that connect two nodes in social networks, they have high "connectivity" in the sense that there are multiple ways for a pair of friends to reach from one to the other.

We calculate the *edge-connectivity* of each pair, and then report the probability distribution, the average $SCos$ with respect to connectivity value and the correlation between connectivity and CN or CC in Fig. S7. From Fig. S7(a) we know that the *edge-connectivity* follows a heterogeneous distribution that the curve of cumulative probability distribution has a very rapid growth, indicating that the values are concentrated within a small range. Figure S7(b) illustrates that higher connectivity correspond to lower similarity. The phenomenon again supports our conclusion that the number, no matter the number of common friends or pathways between two individuals, doesn't play a positive role in increasing the mobility similarity between them. Figure S7(c) and (d) tell that both CN and CC are positively correlated with *edge-connectivity*, i.e., if a pair share more common friends or higher diversity of them, there are more independent pathways between them.

Different from CN and CC which analyze the structure of two individuals' common neighbors, *edge-connectivity* measure social proximity from a different perspective of independent pathways. The formers are local indicators and the latter is a global indicator. Therefore, when we use CN and CC to measure social proximity, we can observe the influence of one metric on mobility similarity when the other one is controlled. However, it's difficult to perform the analysis by combining connectivity with CN or CC in this study.

In summary, we have observed that *edge-connectivity* has no positive influence on mobility similarity, which is similar to CN. Since connectivity is an important metric in graph theory, we will try to investigate the influence of connectivity in social network in the future work.

6 Explorers and returners

In Ref.[13], the authors develop a dichotomy to classify individuals to two profiles, i.e., explorers and returners. Empirical results show that individuals tend to engage in social interactions preferably with the ones of the same profile. Inspired by this research, we try to investigate the correlation between social proximity and mobility similarity of these two distinct kinds of individuals following this methodology.

(1) We firstly calculate the radius of gyration of each individual and their k-radius of gyration (as the radius of gyration computed over the k-th most frequented locations) with k=2,4,8,12,16 and 20.

(2) Then we classify all the individuals to explorer and returner with the bisector method, the same as that in Ref.[13]. When k=4 (or k=8), there are 76,002 (or 59,401) explorers and 25,795 (or 42,396) returners respectively.

(3) Based on the individual profile, we divide all pairs of friends to 3 groups, i.e., explorer-explorer (E-E), explorer-returner (E-R) and returner -returner (R-R).

(4) Within each group, we calculate the average $SCos$ of all pairs. The data size and average $SCos$ in each group are shown in Table S2.

From Table S2 we can observe a consistent phenomenon that the mobility similarity between the same kinds of individuals (E-E and R-R) is obviously higher than that of different kinds (E-R). Consequently, the similar individuals always have similar behavior pattern, especially for the explorers who are interested to wander between various new locations.

Furthermore, we calculate the average $SCos$ with controlling CN or CC in each group when k=8. However, the influence of CN or CC on mobility similarity is unclear due to the small data amount and large standard deviation after grouping. Therefore, more experiments are needed to uncover the correlation between mobility similarity and personal profile.

Table S2: **The data size and average *SCos* in each group**

k	$k=4$			$k=8$		
Group	E-E	E-R	R-R	E-E	E-R	R-R
Pairs of friends	353,842	220,572	43,351	228,561	284,037	105,167
Average SCos	0.0326	0.0275	0.0332	0.0343	0.0282	0.0305

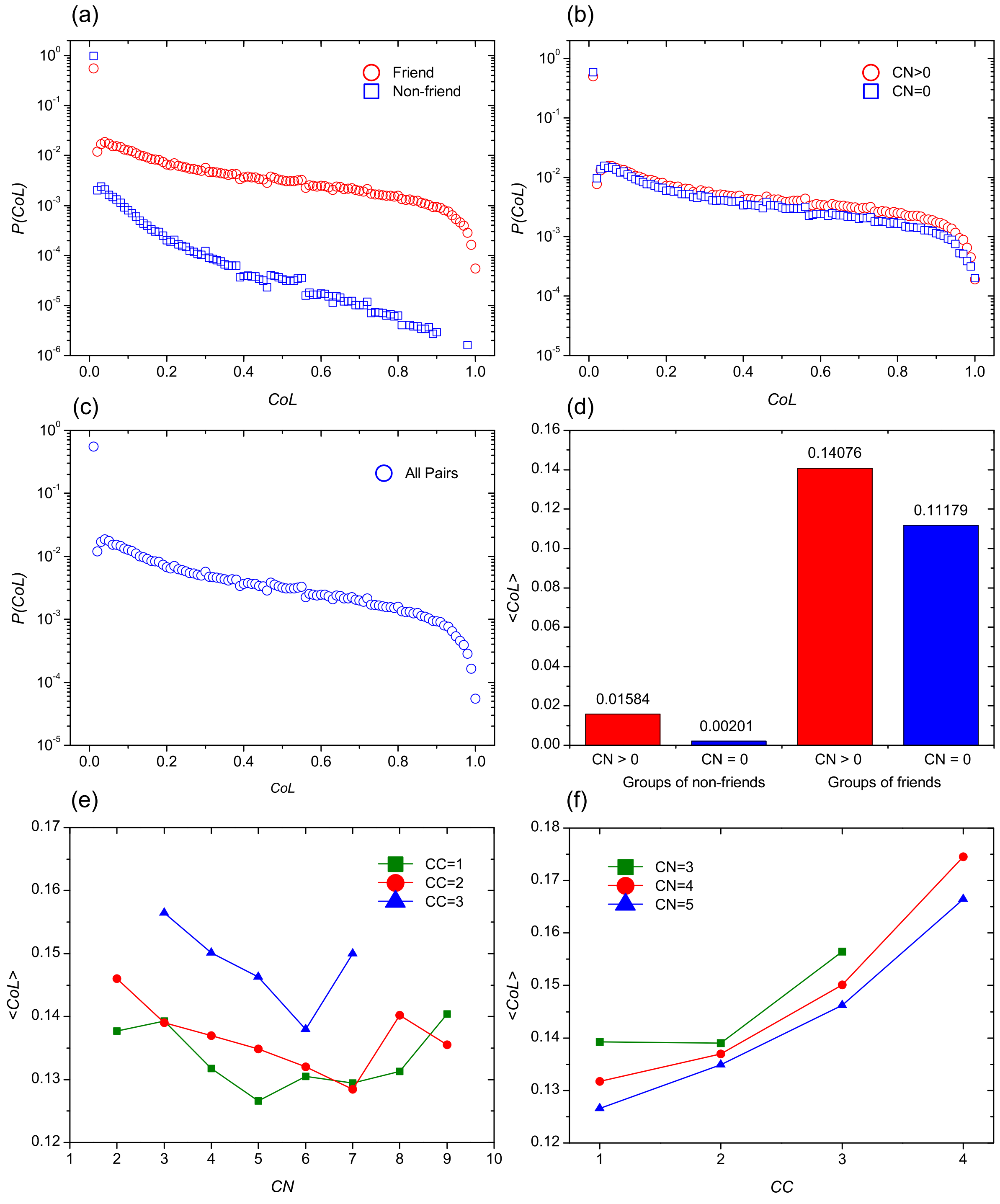

Figure S6: **Correlation between social proximity metrics and mobility similarity Co-Location Rate *(CoL)* between two individuals.** (a) and (b) show the probability distributions of CoL for groups of different types of social relationship, namely, (a) pairs of individuals are or are not friends, and (b) pairs of friends with or without common neighbors (CN). The probability distribution of CoL for all pairs of friends is shown in (c). In (d), the labels above the bars illustrate the average CoL over all pairs of friends in 4 groups, by intersecting the above two factors. (e) and (f) describe the average CoL of samples in different configurations of number of common neighbors and connected components (CC). Samples are grouped by CC (d) and CN (e) respectively.

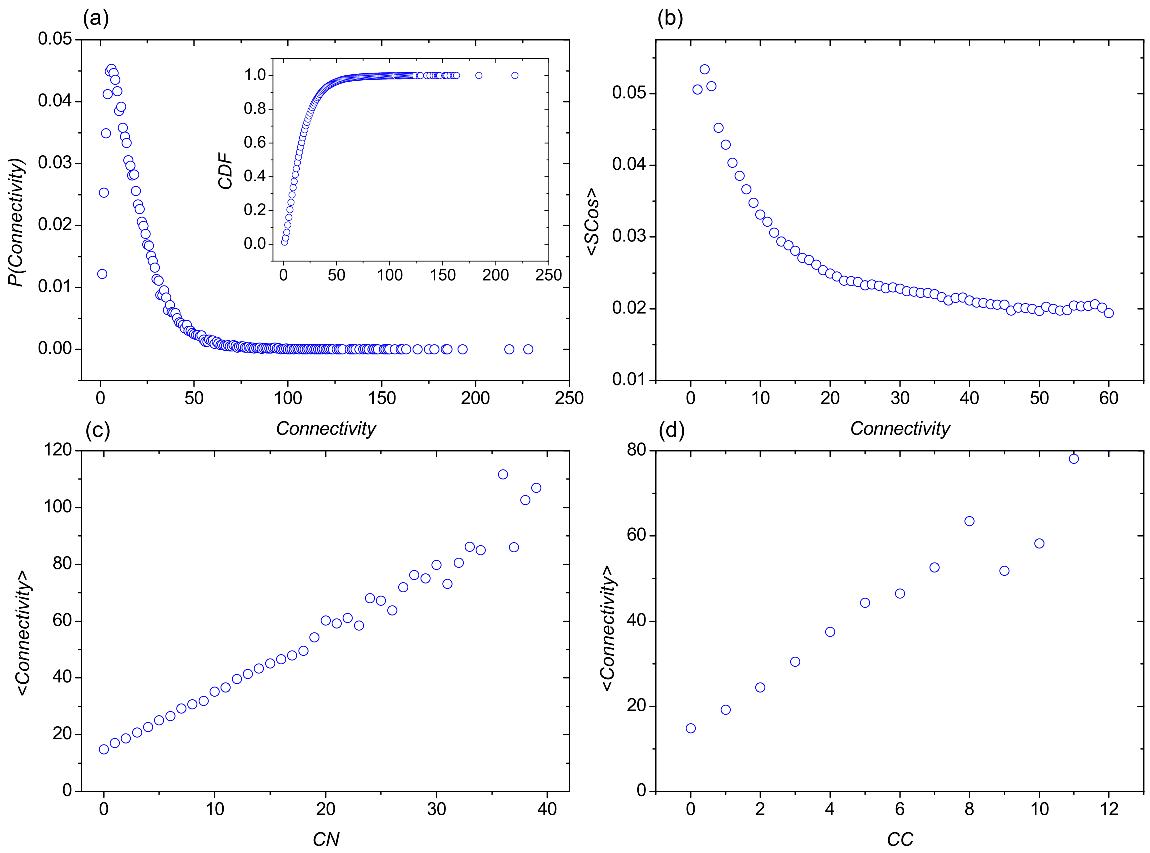

Figure S7: **The relation between *edge-connectivity* and mobility similarity between two individuals.** (a) shows the distribution of *edge-connectivity*, which behaves a right-skewed distribution. The curve peaks at 6 and the average *edge-connectivity* is 17.9. The inset illustrates the cumulative probability distribution (CDF), where the cumulative probability reaches 20.4%, 63.2% or 98.2% when connectivity is 6, 18 or 60. The correlation between the average *edge-connectivity* and number of common neighbors (CN) or the diversity of them (CC) are shown in (c) and (d) respectively, where both of them exhibit positive correlation.
